# CCL2 and PAK6 as Candidate Biomarkers of Neuroinflammation in Parkinson’s Disease: An Integrated Machine Learning and Single-Nucleus Transcriptomic Study

**DOI:** 10.3390/brainsci16050463

**Published:** 2026-04-25

**Authors:** Qixin Zhu, Zhen Zhang, Leiming Zhang, Qian Li, Ting Zhang, Fei Yang

**Affiliations:** 1 Department of Neurobiology, School of Basic Medical Sciences, Capital Medical University, Beijing 100069, China; 122025010032@mail.ccmu.edu.cn (Q.Z.); zhangz@mail.ccmu.edu.cn (Z.Z.); zhang.t@ccmu.edu.cn (T.Z.); 2 Department of Neurosurgery, The Six Medical Center of PLA Hospital, Beijing 100048, China; zhangleiming66@163.com; 3 Department of Biochemistry and Molecular Biology, School of Basic Medical Sciences, Capital Medical University, Beijing 100069, China; qianli@ccmu.edu.cn

**Keywords:** Parkinson’s disease, neuroinflammation, chemokine signaling, immune signatures, CCL2, PAK6, machine learning, single-nucleus RNA sequencing

## Abstract

**Highlights:**

**What are the main findings?**

- CCL2 and PAK6 were prioritized as candidate molecules associated with PD-related neuroinflammatory transcriptional alterations.
- Cross-platform validation and single-nucleus transcriptomic mapping suggested that CCL2 was predominantly associated with astrocytes, whereas PAK6 was more strongly associated with neuronal populations.

**What are the implications of the main findings?**

- The findings support a role for chemokine-associated neuroinflammation in PD and highlight CCL2 and PAK6 as complementary candidate molecules.
- Further experimental validation is needed to determine the biological and translational relevance of CCL2 and PAK6.

**Abstract:**

Background: Neuroinflammation is recognized as a key contributor to Parkinson’s disease (PD), but the relationships between inflammatory signaling, immune-state alterations, and cell-type-specific transcriptional programs remain unclear. Methods: Public transcriptomic datasets, including GSE20141 (discovery cohort) and the substantia nigra subset of GSE114517 (external validation cohort), were analyzed. Genes identified by exploratory differential-expression screening in the discovery cohort were intersected with predefined inflammation- and chemokine-related gene sets to define a candidate space for downstream prioritization. Protein–protein interaction, Gene Ontology, KEGG, and immune-signature analyses were performed, followed by machine learning-based feature prioritization using Elastic Net, support vector machine-recursive feature elimination, and random forest. Prioritized candidates were further evaluated by cross-platform validation, single-nucleus transcriptomic mapping, and a hypothesis-generating in silico perturbation analysis in PD astrocytes. Results: Seventeen genes were retained at the intersection of PD-related differentially expressed genes and inflammation-/chemokine-associated gene sets. These candidates formed a response module enriched in mitochondrial organization, oxidative phosphorylation, and mitophagy pathways. Immune-signature analysis suggested an altered transcriptome-derived immune landscape in PD, with changes in NK cell-related signatures and significant correlations between immune-state scores and the candidate genes. Machine learning-based prioritization yielded five shared candidates, of which only CCL2 and PAK6 showed same-direction support with nominal significance in the external validation cohort. Single-nucleus transcriptomic analysis localized CCL2 predominantly to astrocytes, whereas PAK6 was more strongly associated with neuronal populations, particularly OTX2-positive ventral midbrain neurons. In silico perturbation analysis further predicted that CCL2 suppression in PD astrocytes may be associated with translational- and ribosome-related regulatory programs. Conclusions: CCL2 and PAK6 emerged as prioritized candidate biomarkers associated with PD-related inflammatory and chemokine-linked transcriptional alterations in the substantia nigra. More broadly, this study provides a multi-layered framework for candidate prioritization, cross-platform validation, and cell-type-level contextualization in PD neuroinflammation. Because the study is computational and the perturbation analysis is predictive, orthogonal experimental validation will be required to determine whether CCL2 and PAK6 are biomarkers of disease-associated transcriptional states, functional contributors to PD pathogenesis, or both.

## 1. Introduction

Parkinson’s disease (PD) is the second most common neurodegenerative disorder and a major cause of disability in aging populations worldwide [1,2,3]. Clinically, PD is characterized by bradykinesia, resting tremor, rigidity, postural instability, and a wide range of non-motor symptoms. Neuropathologically, it is defined by progressive loss of dopaminergic neurons in the substantia nigra pars compacta, striatal dopamine depletion, and intracellular aggregates of misfolded α-synuclein in Lewy bodies and Lewy neurites [1,2,3]. Although current treatments, including dopaminergic replacement and deep brain stimulation, can alleviate symptoms, they do not halt the underlying neurodegenerative process [1,3,4]. These limitations have increased interest in molecular pathways that may better reflect disease biology, including neuroinflammatory processes operating in the substantia nigra.

PD has long been studied from the perspective of neuron-intrinsic stress pathways, including mitochondrial dysfunction, impaired proteostasis, and oxidative damage; however, accumulating evidence indicates that neuroinflammation is an important and interacting component of disease pathogenesis [1,4,5]. Both central and peripheral immune abnormalities have been reported in PD, including activation of microglia and astrocytes, altered cytokine networks, and interactions between resident glia and infiltrating immune cells [4,5,6,7,8]. Under physiological conditions, microglia contribute to central nervous system homeostasis, whereas astrocytes support synaptic and metabolic functions; under pathological conditions, both cell types can adopt reactive states that amplify neuronal stress and tissue dysfunction [6,7,8,9,10,11]. Misfolded or extracellular α-synuclein can engage innate immune receptors, including Toll-like receptor 2 and Toll-like receptor 4, thereby promoting pro-inflammatory cytokine release and reactive oxygen species production in glial cells [5,8,12,13,14].

Among these inflammatory mediators, chemokine-related signaling is of particular interest because it regulates cellular chemotaxis and may reshape the neuroimmune microenvironment in PD [4,5,6]. CCL2 is especially relevant in this context: human genetic studies have linked CCL2 polymorphisms to PD susceptibility, and experimental work has shown that CCR2-dependent peripheral monocyte recruitment contributes to α-synuclein-induced inflammation and neurodegeneration [15,16]. By contrast, PAK6 is not a canonical chemokine but a serine/threonine kinase implicated in neuronal regulatory pathways, including LRRK2-associated signaling, and recent clinical work has suggested that plasma PAK6 may have biomarker potential in PD [17,18]. Together, these observations suggest that candidate molecules linked to PD neuroinflammation may capture not only chemokine-associated immune remodeling but also neuron-associated regulatory vulnerability.

Because PD progression is heterogeneous and involves multiple interacting cell populations, conventional single-gene approaches are often insufficient to capture the complexity of disease-associated molecular programs [1,19]. Public transcriptomic resources, together with bioinformatics workflows, have therefore become valuable tools for identifying candidate biomarkers and disease-relevant pathways in PD [19,20]. However, bulk-tissue transcriptomic studies alone provide limited cell-type resolution, whereas single-cell and single-nucleus transcriptomic approaches can assign candidate genes to defined cellular compartments and help distinguish neuronal from glial transcriptional alterations in diseased tissue [21,22]. Notably, recent studies have already applied related bioinformatics and machine learning strategies to inflammatory biomarker discovery in PD, highlighting the need to position new analyses as focused extensions or contextualization efforts rather than as entirely new inflammatory frameworks [20].

In the present study, we applied an integrated workflow combining exploratory differential-expression screening, functional enrichment analysis, immune-signature profiling, machine learning-based prioritization, cross-platform validation in GSE114517, and single-nucleus transcriptomic mapping to prioritize candidate molecules associated with PD-related neuroinflammation. Rather than seeking to establish definitive causal mechanisms, we aimed to identify and contextualize candidates within relevant cellular compartments and to use in silico perturbation as a hypothesis-generating extension in disease-relevant glial populations. Using this strategy, we prioritized CCL2 and PAK6 as two candidates that may reflect complementary aspects of PD biology, namely chemokine-associated immune remodeling and neuron-associated regulatory vulnerability.

## 2. Materials and Methods

### 2.1. Study Design

This study was designed as an integrative, public-dataset-based workflow to prioritize candidate molecules associated with PD-related neuroinflammation. GSE20141 was used as the discovery cohort for exploratory screening of inflammation- and chemokine-related differentially expressed genes. These candidates were then characterized by functional enrichment and immune-signature analyses, followed by machine learning-based prioritization. The prioritized genes were further evaluated in the independent substantia nigra validation cohort GSE114517. To provide cell-type-level context, the final prioritized candidates were mapped onto single-nucleus RNA-sequencing (snRNA-seq) data from human midbrain tissue (GSE253462). Finally, because CCL2 showed strong astrocyte-associated expression, an in silico perturbation analysis was performed in PD astrocytes as a hypothesis-generating step.

### 2.2. Ethical Statement

This study analyzed only publicly available, de-identified datasets obtained from the Gene Expression Omnibus (GEO) database (GSE20141, GSE114517, GSE148434, GSE253462, and GSE253975). No new human samples or identifiable personal data were collected or used. Therefore, additional ethical approval and informed consent were not required for this study.

### 2.3. Data Acquisition and Preprocessing

Bulk transcriptomic datasets derived from the post-mortem substantia nigra tissue of patients with PD and neurologically normal controls were retrieved from the Gene Expression Omnibus (GEO) database [23]. The GSE20141 dataset, generated on the GPL570 platform, comprised expression profiles from substantia nigra tissue obtained from 10 PD cases and 8 controls and served as the discovery cohort [23]. GSE114517, generated on the GPL18573 platform, included ribosomal-RNA-depleted RNA-seq libraries from post-mortem human brain tissue; only the substantia nigra samples were retained for this study, yielding 17 PD and 12 control samples for external validation.

For the discovery cohort, raw or processed microarray expression data were background-corrected, log2(x + 1) transformed, and quantile-normalized using the limma::normalizeBetweenArrays function (method = ‘quantile’) [24]. For the validation cohort, the substantia nigra subset of GSE114517 was retained, and the author-provided supplementary PD-versus-control differential-expression table (GSE114517_Diff_PDvsCont.csv.gz), generated with edgeR, was used as the primary external validation reference for directionality and nominal significance [25].

To define the candidate gene space for inflammation- and chemokine-associated screening, genes from the Molecular Signatures Database (MSigDB) inflammatory response collection were combined with a chemokine-related gene list curated before analysis [26]. Publicly available snRNA-seq data from post-mortem human midbrain tissue (GSE253462) were used for cell-type-specific contextualization of the final prioritized candidates [22]. For supplementary external support, we additionally examined the bulk RNA-seq component of GSE148434 and the spatial transcriptomic dataset GSE253975. GSE148434 was used to provide supplementary visualization of differential-expression patterns in an independent human substantia nigra dataset, whereas GSE253975 was used to visualize the spatial distribution of CCL2 across control and PD substantia nigra samples.

### 2.4. Differential Expression Analysis and Candidate Gene Identification

Differentially expressed genes (DEGs) between PD and control samples in the GSE20141 training cohort were identified using limma (lmFit and eBayes) (version 3.66.0) [24]. Genes meeting the predefined significance thresholds of |log2FC| ≥ 1 and nominal *p* < 0.05 were retained as differentially expressed candidates for downstream exploratory prioritization. Given the limited sample size and the exploratory aim of discovery-stage screening, we used nominal *p* values together with effect-size filtering to define a candidate gene set for subsequent prioritization and independent validation. Candidate genes were then defined as the intersection between the training-set DEGs and the union of inflammation-related and chemokine-related gene sets. Heatmaps, volcano plots, and intersection plots were generated to visualize the screening workflow. These visualizations were generated in R (version 4.4.3) using ggplot2-based workflows and related plotting packages (version 4.0.2).

### 2.5. Protein–Protein Interaction Network and Functional Enrichment Analyses

To assess the functional relationships among the intersecting candidate genes, a protein–protein interaction (PPI) network was constructed using the STRING database (combined score cutoff = 0.4) and visualized in Cytoscape (version 3.10.4) [27,28]. Functional enrichment analyses were then performed using clusterProfiler (version 4.14.0) in R [29]. Specifically, GO enrichment was conducted with enrichGO across the Biological Process (BP), Cellular Component (CC), and Molecular Function (MF) categories, and KEGG enrichment was conducted with enrichKEGG, using Benjamini–Hochberg correction (pAdjustMethod = “BH”). The top enriched terms and pathways were visualized according to their adjusted *p* values [29]. For display, the top 10 terms from each GO (BP, CC, and MF) and the top 10 KEGG pathways were shown. GO and KEGG enrichment plots were generated in R using clusterProfiler and ggplot2-based visualization functions.

### 2.6. Immune Signature Analysis

Immune-related signatures in the GSE20141 discovery cohort were quantified using the gene set variation analysis (GSVA) package (version 2.4.4) with the single-sample Gene Set Enrichment Analysis (ssGSEA) method. Specifically, ssGSEA scores were calculated using GSVA::ssgseaParam with alpha = 0.25 and normalize = TRUE, based on predefined immune-cell signature gene sets [30]. Relative immune-signature levels were compared between PD and control groups using the Wilcoxon rank-sum test. Spearman correlation analysis among immune signatures and between immune signatures and the candidate genes was performed using Hmisc::rcorr (version 5.2–5). Correlations with |r| > 0.3 and *p* < 0.05 were highlighted in the correlation heatmaps. The ssGSEA results were interpreted as transcriptome-derived immune-state signatures rather than direct measurements of immune-cell abundance or histological infiltration. Accordingly, these analyses were used primarily to explore disease-associated immune-related transcriptional patterns and their correlations with candidate genes.

### 2.7. Machine Learning-Based Candidate Prioritization and External Validation

To prioritize candidate genes, three complementary machine learning algorithms were applied in R to the 17 intersecting candidate genes: Elastic Net using the glmnet package (version 4.1–10), support vector machine-recursive feature elimination (SVM-RFE) using e1071 (version 1.7–17), and random forest using randomForest (version 4.7–1.2) [31,32]. For Elastic Net (seed = 1), five-fold stratified cross-validation was performed using cv.glmnet with alpha = 0.1, and genes retained at lambda.1se were selected. For SVM-RFE (seed = 88), a linear-kernel SVM was trained with five-fold stratified cross-validation, and the top 7 features yielding the highest balanced accuracy were retained. For random forest (seed = 2), ntree = 1000 was used during mtry tuning, followed by a final model with ntree = 2000 and the best mtry (best_mtry = 2). The final machine learning candidates were defined as the intersection of the three algorithms. External validation was performed using the author-provided GSE114517 differential-expression table. Only genes showing the same direction of change in the training and validation cohorts and nominal *p* < 0.05 in the validation cohort were retained as final prioritized candidates. Receiver operating characteristic (ROC) analysis was subsequently performed using the pROC package (version 1.19.0.1) in the external validation cohort to assess the discriminatory performance of the retained prioritized candidates, and the area under the curve (AUC) was used to quantify classification accuracy. Boxplots and ROC curves were generated in R using ggplot2 and pROC.

### 2.8. Gene Set Enrichment Analysis and Gene Interaction Network Analysis of Final Prioritized Candidates

To further characterize the biological programs associated with the final prioritized candidates, Gene Set Enrichment Analysis (GSEA) was performed based on gene correlations [33]. For each prioritized candidate, genes in the training cohort were ranked according to their Spearman correlation with the candidate using psych::corr.test (method = “spearman”, adjust = “none”) (version 2.6.3). The resulting ranked gene list was analyzed using clusterProfiler::GSEA with parameters set to minGSSize = 10, maxGSSize = 500, and pvalueCutoff = 1. Pathways with an absolute normalized enrichment score (|NES|) > 1, nominal *p* < 0.05, and false discovery rate (FDR) < 0.25 were considered significant. The top 10 enriched pathways for each prioritized candidate were visualized.

To explore the broader interaction context of the final prioritized candidates, gene interaction networks centered on CCL2 and PAK6 were constructed using GeneMANIA [34]. The GSEA plots were generated in R, and the interaction networks were produced using the GeneMANIA web platform. These networks were used for descriptive interpretation of genes potentially linked to chemokine-associated inflammatory signaling, neuronal regulatory processes, or broader PD-related molecular programs.

### 2.9. snRNA-Sequencing Analysis

Public snRNA-seq data from post-mortem human midbrain tissue (GSE253462) were analyzed using the Seurat package (version 5.4.0) in R [35]. Low-quality nuclei were excluded according to predefined quality-control criteria (nFeature_RNA < 200 or > 2500, nCount_RNA < 500 or > 2443, percent.mt > 5, and percent.hb > 2). After quality control, SCTransform normalization was performed using method = ‘glmGamPoi’, variable.features.n = 3000, and vst.flavor = ‘v2’. Principal component analysis (PCA) was run with 50 components, followed by neighborhood construction with dims = 1:30, clustering with resolution = 0.5, and Uniform Manifold Approximation and Projection (UMAP) visualization with dims = 1:30 [35,36]. Cluster marker genes were identified from the RNA assay after NormalizeData and JoinLayers using FindAllMarkers with only.pos = TRUE, min.pct = 0.25, logfc.threshold = 0.25, test.use = ‘wilcox’, and max.cells.per.ident = 200. Cell clusters were annotated according to canonical marker genes for major central nervous system cell populations, including astrocytes, oligodendrocytes, microglia, activated microglia, border-associated macrophage (BAM)-like cells, endothelial cells, GABAergic neurons, dopaminergic neurons, oligodendrocyte precursor cells (OPCs), and OTX2-positive ventral midbrain neurons. UMAP plots were generated in Seurat.

The expression of the final prioritized candidates was then mapped across annotated cell populations. Cell-type fractions were compared between PD and control groups at the sample level using the Wilcoxon rank-sum test. Sample-level cell-type composition was visualized using SCpubr::do_BarPlot (version 3.0.1), and functional enrichment of the differential cell type was examined using ReactomeGSA (version 1.20.0) with the ssGSEA method. Because astrocytes showed the clearest compositional difference between groups in the single-nucleus dataset, they were selected for downstream communication, trajectory, metabolic, and perturbation analyses.

Astrocytes were subsetted and re-clustered before trajectory analysis. In the astrocyte subset, NormalizeData, FindVariableFeatures (nfeatures = 2000), ScaleData, RunPCA (npcs = 30), FindNeighbors (dims = 1:20), FindClusters (resolution = 0.4), and RunUMAP (dims = 1:20) were performed. Pseudotime trajectories were reconstructed using Monocle2 (version 2.34.0). A CellDataSet was created from RNA counts, followed by estimateSizeFactors, estimateDispersions, reduceDimension (method = ‘DDRTree’), and orderCells. Genes associated with pseudotime were identified using differentialGeneTest with fullModelFormulaStr = ‘~sm.ns(Pseudotime)’. Dynamic genes were visualized using plot_pseudotime_heatmap with num_clusters = 4, and GO BP enrichment analysis was performed using clusterProfiler.

Cell–cell communication analysis between control and PD conditions was performed using the CellChat package (version 2.2.0.9001). Astrocytes were treated as the key cell type, and astrocyte-associated ligand-receptor interactions, interaction counts, and interaction strengths were compared between the two conditions. Furthermore, metabolic pathway activity in the key cell type was assessed using the scMetabolism package (version 0.2.1) with the AUCell backend. Cell-type composition plots, trajectory visualizations, and related single-nucleus figures were generated using Seurat, SCpubr, and Monocle2.

### 2.10. In Silico CCL2 Perturbation Analysis in PD Astrocytes

To generate hypotheses regarding the potential regulatory consequences of CCL2 suppression in the disease-relevant glial compartment, an in silico perturbation analysis was performed in PD astrocytes using the scTenifoldKnk package (version 1.0.3). RNA-count data from PD astrocytes were extracted, and genes were prefiltered to a 2500-gene input matrix. scTenifoldKnk was then run with gKO = ‘CCL2’, qc = FALSE, nc_nNet = 10, nc_nCells = min(500, ncol(countMatrix)), nc_nComp = 3, td_K = 3, ma_nDim = 2, and nCores = 1. Differentially regulated genes after simulated CCL2 perturbation were ranked according to the scTenifoldKnk differential-regulation statistics, and GO BP and KEGG enrichment analyses were performed to characterize the predicted biological effects of CCL2 perturbation in PD astrocytes.

### 2.11. Statistical Analysis

All analyses were performed in R version 4.4.3. Differential-expression analysis in the discovery cohort was performed with limma. Because no genes passed the FDR threshold in the training cohort, nominal *p* values together with effect-size filtering were used only for exploratory candidate screening and prioritization, and all downstream interpretations were made cautiously in conjunction with external validation and cell-type-level contextualization. Wilcoxon rank-sum tests were used for immune-signature comparisons and sample-level cell-type-fraction comparisons. Spearman correlation analyses were performed using Hmisc::rcorr where applicable. For GSEA, pathways with |NES| > 1, nominal *p* < 0.05, and FDR < 0.25 were considered significant unless otherwise specified. A two-sided *p* value < 0.05 was considered statistically significant unless otherwise stated.

## 3. Results

### 3.1. Identification of Inflammation- and Chemokine-Associated Differentially Expressed Genes

**Figure 1:**
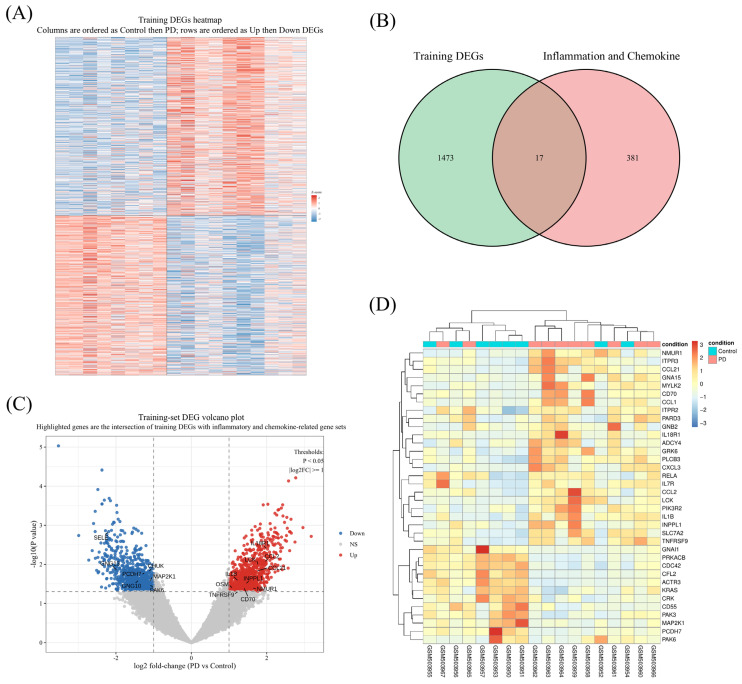
Differentially expressed genes and inflammation/chemokine-associated candidate genes in the training cohort. (**A**) Heatmap of training-set differentially expressed genes (DEGs); columns are ordered as control followed by Parkinson’s disease (PD), and rows are ordered as upregulated followed by downregulated DEGs. (**B**) Venn diagram showing the overlap between training-set DEGs and inflammation- and chemokine-related genes, yielding 17 intersecting candidates. (**C**) Volcano plot of the training-set DEGs; genes belonging to the intersection shown in panel B are highlighted. The vertical dashed lines indicate |log2FC| = 1, and the horizontal dashed line indicates nominal *p* = 0.05. (**D**) Heatmap of the 17 intersecting genes across the control and PD samples.

Differential-expression analysis of GSE20141 identified 1490 genes that met the preset criteria for significance, and the global heatmap showed broad transcriptional differences between control and PD samples (Figure 1A). Intersecting these DEGs with the curated inflammation/chemokine gene set yielded 17 overlapping genes (Figure 1B). The volcano plot showed that 10 of the 17 intersecting genes were upregulated and 7 were downregulated in PD (Figure 1C). A focused heatmap of these 17 genes further demonstrated a coherent disease-associated pattern, with higher expression of genes such as CCL2, IL1β, TNFRSF9, and NMUR1 and lower expression of genes including PAK6, GNG10, and PCDH7 in PD samples (Figure 1D). Together, these results narrowed the analysis from global transcriptional change to a focused inflammatory/chemokine candidate set for downstream investigation.

### 3.2. Immune-Signature Analysis Revealed a Shifted Immune Landscape in PD

**Figure 2:**
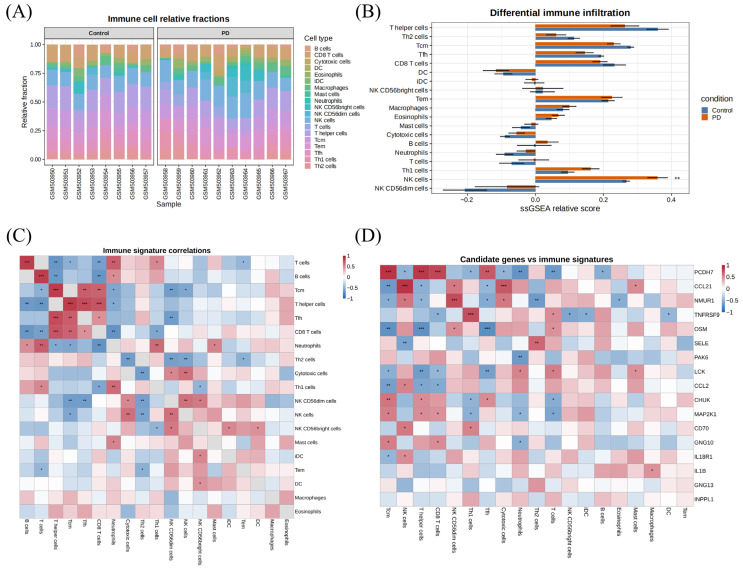
Immune-signature analysis in the training cohort. (**A**) Stacked bar plots showing single-sample Gene Set Enrichment Analysis (ssGSEA)-derived relative immune-signature fractions in control and PD samples. (**B**) Comparison of inferred immune-signature levels between groups. (**C**) Correlation matrix among immune-cell signatures. (**D**) Correlation heatmap between the 17 inflammation/chemokine-associated genes and inferred immune-cell signatures. Asterisks indicate statistical significance (Wilcoxon * *p* < 0.05, ** *p* < 0.01, *** *p* < 0.001).

ssGSEA-based immune-signature profiling revealed an altered immune landscape in PD. The stacked relative-fraction plots showed a redistribution of multiple inferred immune populations between control and PD samples (Figure 2A). Groupwise comparison indicated that the natural killer (NK) cell-related signature differed significantly between the two groups, whereas several additional lymphoid and myeloid signatures showed directional shifts that did not reach statistical significance (Figure 2B). Correlation analysis across immune signatures demonstrated a structured immune network, with both positive and negative associations among lymphoid and myeloid compartments rather than random fluctuations (Figure 2C). When the 17 intersecting genes were correlated with inferred immune-cell fractions, several candidates showed consistent associations with T-cell-, NK-cell-, and myeloid-related signatures (Figure 2D), supporting the interpretation that these genes were embedded in a disease-altered neuroimmune environment.

### 3.3. Interaction Network Construction and Functional Enrichment Analysis of the Selected Candidates

**Figure 3:**
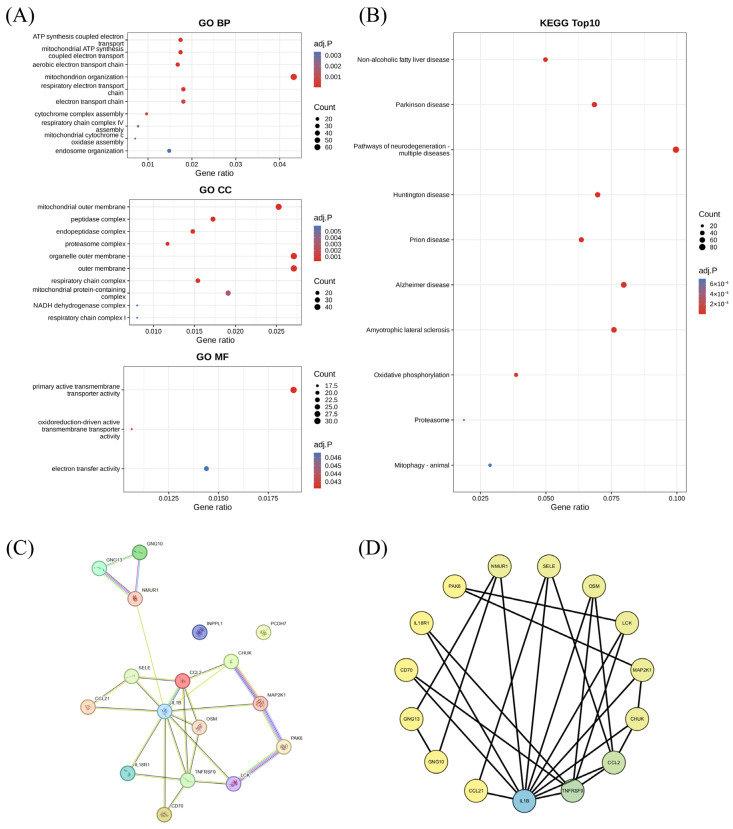
Protein–protein interaction and functional enrichment analyses of the 17 intersecting genes. (**A**) Gene Ontology (GO) enrichment results for biological process (BP), cellular component (CC), and molecular function (MF). (**B**) Top enriched Kyoto Encyclopedia of Genes and Genomes (KEGG) pathways. (**C**) STRING-derived protein–protein interaction (PPI) network. Nodes represent proteins encoded by the input genes, and edges represent functional associations supported by different evidence sources, including curated databases, experimental evidence, co-expression, text mining, gene neighborhood, gene fusion, gene co-occurrence, and protein homology. Node colors are used by STRING for network visualization and do not indicate differential expression or functional categories. (**D**) Degree-based Cytoscape visualization of the candidate-gene PPI network. Nodes represent proteins encoded by the candidate genes, and edges represent STRING-based functional associations between proteins. Node colors reflect degree centrality.

Functional enrichment analysis first highlighted the biological context of the 17 intersecting genes. GO analysis showed enrichment in mitochondrial organization, electron transport, respiratory-chain assembly, and endosome-related processes (Figure 3A), while KEGG analysis identified PD, pathways of neurodegeneration, oxidative phosphorylation, proteasome, and mitophagy among the most enriched pathways (Figure 3B).

To further define the interaction structure of these genes, a PPI network was constructed using STRING and Cytoscape (Figure 3C,D). IL1β, CCL2, and TNFRSF9 occupied central positions in the network, with additional connections involving IL18R1, LCK, CHUK, MAP2K1, and related regulators, indicating that the overlap genes formed an interconnected response module rather than a set of isolated hits. These data suggest that the inflammatory/chemokine signature identified here was coupled to mitochondrial and proteostatic pathways that are closely linked to PD pathobiology.

### 3.4. Machine Learning Prioritization Narrowed the Candidate Set and Identified Genes with External Support

**Figure 4:**
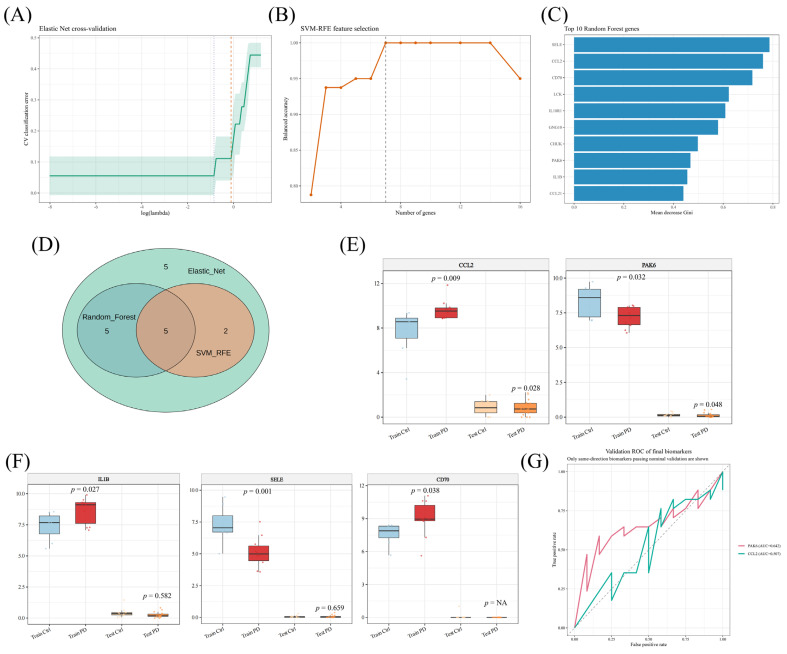
Machine learning-based biomarker screening and external validation. (**A**) Elastic Net cross-validation plot used for feature selection. The green line represents the mean cross-validation error, and the shaded green area indicates the corresponding error range. The grey and orange dashed vertical lines indicate the optimal lambda values selected using the minimum-error criterion and the one-standard-error criterion, respectively. (**B**) Support vector machine-recursive feature elimination (SVM-RFE) feature-selection performance across different gene numbers. The orange line represents model performance, and the grey dashed vertical line indicates the selected optimal number of genes. (**C**) Top-ranked genes identified by random forest according to Mean Decrease Gini. Longer blue bars indicate higher feature importance in the random forest model. (**D**) Venn diagram showing the overlap among Elastic Net, SVM-RFE, and random forest results, identifying five shared candidates. Numbers inside the circles indicate the number of genes in each overlap category. (**E**) Boxplots showing expression of CCL2 and PAK6 in the GSE20141 discovery cohort and the GSE114517 validation cohort. (**F**) Boxplots showing IL1β, SELE, and CD70 expression in the discovery and validation cohorts. (**G**) Receiver operating characteristic (ROC) curves for the final prioritized candidates retained after cross-dataset same-direction validation. The dashed diagonal indicates chance-level discrimination.

Three complementary feature-selection algorithms were applied to the 17 intersecting genes. Elastic Net cross-validation identified a compact solution near the minimum classification error (Figure 4A), SVM-RFE achieved its best performance with a limited number of genes (Figure 4B), and random forest ranked SELE, CCL2, CD70, LCK, IL18R1, GNG10, CHUK, PAK6, IL1β, and CCL21 as the top informative variables by Mean Decrease Gini (Figure 4C). Intersection of the three algorithms yielded five shared candidates—CCL2, PAK6, IL1β, SELE, and CD70—which were carried forward for external evaluation (Figure 4D).

In the discovery cohort, all five genes showed differential expression. In the independent GSE114517 substantia nigra RNA-seq cohort, however, only CCL2 and PAK6 retained both nominal significance and the same direction of change observed in GSE20141 (Figure 4E). Specifically, CCL2 remained upregulated in PD, whereas PAK6 remained downregulated. By contrast, IL1β lost statistical support in the validation dataset, SELE showed discordant directionality between cohorts, and CD70 lacked robust validation support (Figure 4F). ROC analysis was therefore restricted to the genes that passed same-direction validation, and both CCL2 and PAK6 showed modest but above-chance discriminatory performance in the external dataset (Figure 4G). To further examine feature-selection stability across random initializations, we also summarized the per-seed retention pattern of CCL2 across 0–100 random seeds; CCL2 showed high cross-seed stability across methods, with only rare misses in SVM-RFE (Supplementary Figure S1). As an additional external reference, a supplementary volcano plot from GSE148434 further illustrated the PD-upregulated position of CCL2 in an independent substantia nigra dataset (Supplementary Figure S2A).

### 3.5. Single-Nucleus Analysis Localized Candidate-Gene Expression to Distinct Cellular Compartments

**Figure 5:**
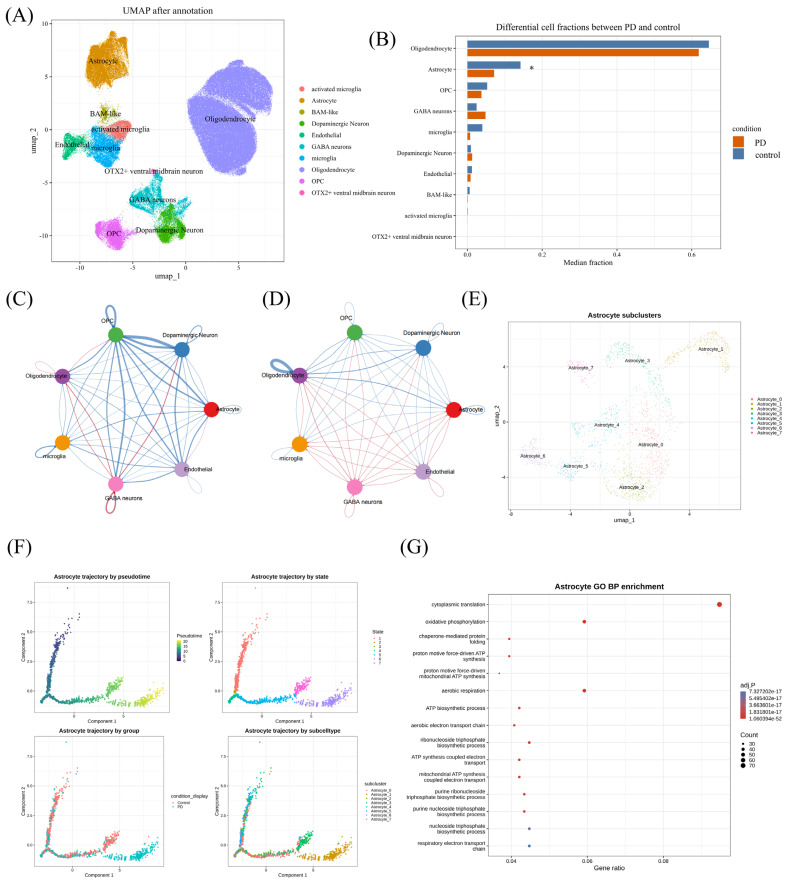
Single-nucleus transcriptomic, cell–cell communication, and astrocyte trajectory analyses. (**A**) Uniform Manifold Approximation and Projection (UMAP) showing the annotated cell populations. (**B**) Comparison of median cell fractions between PD and control groups. Asterisk indicates Wilcoxon *p* < 0.05. (**C**) Differential interaction-count network between control and PD conditions, showing changes in the number of cell–cell communications. (**D**) Differential interaction-weight network between control and PD conditions, showing changes in communication strength. (**E**) UMAP of astrocyte subclusters after reclustering, resolving Astrocyte_0 to Astrocyte_7. (**F**) Pseudotime panels showing the astrocyte trajectory colored by pseudotime, state, group, and subcluster. (**G**) GO biological process (GO BP) enrichment of genes associated with astrocyte pseudotime.

Single-nucleus analysis was first used to localize candidate-gene expression to specific cellular compartments. UMAP annotation resolved the dataset into astrocytes, oligodendrocytes, microglia, activated microglia, BAM-like cells, endothelial cells, GABA neurons, dopaminergic neurons, OPCs, and OTX2-positive ventral midbrain neurons (Figure 5A). Marker visualization supported the assigned identities across clusters (Supplementary Figure S3A). When the two prioritized genes were projected onto the annotated cell atlas, CCL2 expression was concentrated mainly in astrocytes, whereas PAK6 expression was strongest in OTX2-positive ventral midbrain neurons and was also detectable in other neuronal populations (Supplementary Figure S3B). Differential expression at the cell-type level further showed increased astrocytic CCL2 in PD relative to control, whereas PAK6 signal within astrocytes remained minimal (Supplementary Figure S3C). Consistent with this astrocyte-associated signal, spatial transcriptomic data from GSE253975 showed sparse CCL2-positive spots in control samples but broader and more frequent CCL2 expression in several PD substantia nigra samples, providing spatial support for disease-associated CCL2 upregulation (Supplementary Figure S2B). Compositional analysis identified astrocytes as the differential cell type, with a significantly lower astrocyte fraction in PD compared to control samples (Wilcoxon *p* = 0.0218; Figure 5B; Supplementary Figure S3D). Because astrocytes were the only cell type showing a significant compositional difference between PD and control, they were selected for downstream communication, trajectory, metabolic, and perturbation analyses.

CellChat-based comparison further revealed broad remodeling of intercellular communication patterns between control and PD conditions. Differential interaction networks showed changes in both the number and strength of cell–cell communications, including astrocyte-associated interactions (Figure 5C,D). To investigate astrocyte heterogeneity in more detail, astrocytes were reclustered into eight subclusters, designated Astrocyte_0 to Astrocyte_7 (Figure 5E). Pseudotime analysis arranged astrocytes along a structured trajectory, with cells distributed across different pseudotime positions, states, conditions, and subcluster-defined branches (Figure 5F). Genes associated with this astrocyte trajectory were enriched in biological processes related to oxidative phosphorylation, aerobic respiration, respiratory electron transport, ATP synthesis-coupled electron transport, nucleoside triphosphate biosynthesis, and chaperone-mediated protein folding (Figure 5G). Detailed astrocyte-associated ligand–receptor pairs that were relatively downregulated or upregulated in PD, together with summary comparisons of overall interaction counts and interaction weights, are shown in Supplementary Figure S4. Together, these analyses suggest that PD-associated astrocyte remodeling involves quantitative compositional change, altered intercellular communication, and state transitions.

### 3.6. Network-Context Analysis and GSEA Highlighted Distinct Biological Associations for PAK6 and CCL2

**Figure 6:**
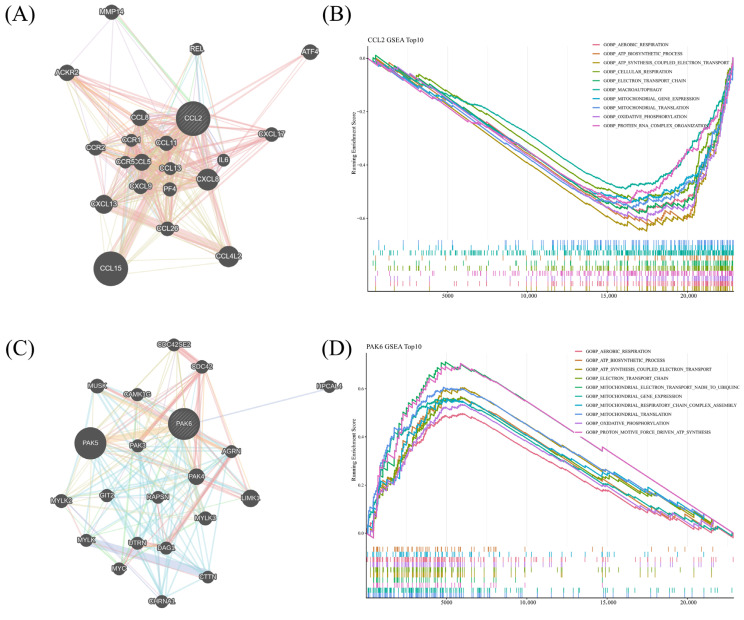
Network-context analysis and gene set enrichment analysis (GSEA) of the final prioritized candidates. (**A**) GeneMANIA-based functional association network centered on CCL2. (**B**) Top 10 GSEA results associated with CCL2. (**C**) GeneMANIA-based functional association network centered on PAK6. (**D**) Top 10 GSEA results associated with PAK6. In the GeneMANIA networks, nodes represent genes or their encoded proteins, and edges represent functional associations inferred from multiple evidence sources. Edge colors indicate different types of supporting evidence, including co-expression, physical interactions, pathway associations, co-localization, genetic interactions, predicted interactions, and shared protein domains. Larger nodes indicate genes with greater inferred relevance within the GeneMANIA-generated network.

Because CCL2 and PAK6 showed the most consistent support across datasets, they were selected for extended downstream analyses. Network-context mapping placed CCL2 within a chemokine-centered inflammatory network connected to CCL5, CCL13, CCL19, CCL26, CCR1, CCR2, CCR5, CXCL8, and IL6 (Figure 6A), whereas PAK6 localized to a module enriched for cytoskeletal, synaptic, and membrane-associated interactors such as PAK3, PAK5, LIMK1, DAG1, RAPSN, and AGRN (Figure 6C).

GSEA further indicated that CCL2-stratified transcriptional variation showed reciprocal associations with aerobic respiration, ATP biosynthesis, ATP synthesis-coupled electron transport, cellular respiration, and oxidative phosphorylation (Figure 6B). By contrast, PAK6-associated transcriptional variation was linked to aerobic respiration, ATP biosynthetic process, mitochondrial organization, respiratory-chain function, oxidative phosphorylation, and protein/RNA complex organization (Figure 6D). These results suggest that CCL2 and PAK6 represent two linked but biologically distinct axes of PD-associated dysregulation: one centered on chemokine-driven inflammatory activation and the other more closely aligned with neuronal and metabolic integrity.

### 3.7. In Silico CCL2 Perturbation Suggested Predicted Regulatory Changes in PD Astrocytes

**Figure 7:**
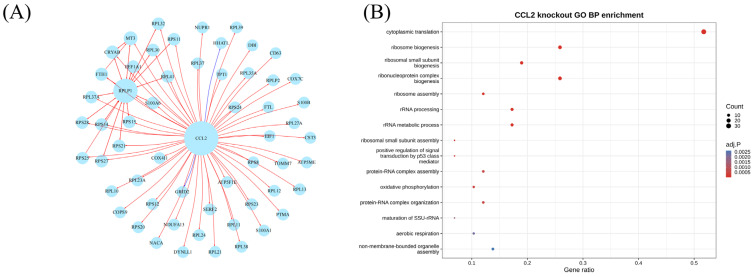
Interaction network and functional enrichment analyses of genes altered after in silico astrocytic CCL2 perturbation. (**A**) CCL2-centered interaction network of genes differentially regulated after simulated astrocytic CCL2 perturbation. Red nodes indicate genes predicted to be upregulated after perturbation, whereas blue nodes indicate genes predicted to be downregulated. (**B**) GO biological process (GO BP) enrichment analysis of genes predicted to be upregulated after astrocytic CCL2 perturbation, highlighting translation- and ribosome biogenesis-related biological processes.

Because CCL2 showed robust cross-dataset support and was strongly enriched in astrocytes (Supplementary Figure S3B,C), we next performed an in silico CCL2 perturbation analysis in PD astrocytes using scTenifoldKnk (version 1.0.3). Using 8249 PD astrocytes and a 2500-gene input matrix, scTenifoldKnk identified 58 significantly differentially regulated genes at FDR < 0.05 after simulated CCL2 perturbation (Figure 7A). Enrichment analysis of these differential regulation signals highlighted pathways related to cytoplasmic translation, ribosome biogenesis, and related biosynthetic programs (Figure 7B). Together, these results suggest that CCL2-associated perturbation may be linked to translational- and ribosome-related regulatory programs in PD astrocytes.

## 4. Discussion

In the present study, we applied an integrated transcriptomic workflow to prioritize inflammation- and chemokine-associated candidate molecules in PD and identified CCL2 and PAK6 as the most informative prioritized candidates after machine learning-based prioritization, external validation, and single-nucleus transcriptomic mapping. Together, our findings suggest that PD-associated transcriptional alterations in the substantia nigra reflect coordinated interactions among chemokine-associated immune remodeling, mitochondrial dysfunction, and cell-type-specific vulnerability, rather than a single inflammatory axis alone.

Although our study shares the discovery dataset GSE20141 with the recent work by Li et al., the two studies differ in analytical focus and interpretive scope. Li et al. primarily examined inflammation-related genes and identified candidate biomarkers through machine learning analysis with external validation [20,37,38]. In contrast, our study focused on the intersection between PD-related differential expression and inflammation- and chemokine-associated genes, followed by cross-platform validation in an independent substantia nigra RNA-seq cohort and single-nucleus transcriptomic contextualization. Accordingly, the present work is better viewed as a biologically focused extension within a chemokine-associated neuroimmune framework rather than a direct replication of prior inflammation-centered biomarker studies.

Among the two prioritized genes, CCL2 is particularly notable because it links the bulk transcriptomic findings, immune-signature analysis, and single-nucleus results within a coherent neuroinflammatory context. CCL2 is broadly implicated in central nervous system inflammation and has been reported in astrocytes, microglia, and neurons [15]. Experimental studies further indicate that the CCL2/CCR2 axis is mechanistically relevant to PD-related neuroinflammation, as CCR2-dependent peripheral monocyte recruitment contributes to α-synuclein-induced inflammatory amplification and neurodegeneration [16]. Human studies likewise support associations between CCL2-related genetic variation, peripheral CCL2 alterations, and PD susceptibility or disease-related inflammatory activity [15,39]. In our study, however, the single-nucleus data suggested that the strongest CCL2-associated signal was concentrated in astrocytes, supporting the interpretation that astrocytes may represent an important cellular context for chemokine-associated inflammatory communication in PD [11,38]. In addition, our in silico CCL2 perturbation analysis generated the hypothesis that astrocytic CCL2 may be linked to translational- and ribosome-associated regulatory programs. This observation suggests that, beyond its canonical role in extracellular monocyte recruitment, CCL2-associated signaling may also be related to intracellular biosynthetic and proteostatic states within reactive astrocytes. However, these predicted relationships remain computational and will require orthogonal experimental validation. Taken together, these findings suggest that CCL2 may serve as a transcriptional readout of a glia-centered inflammatory program that could contribute to shaping the local immune microenvironment in the substantia nigra.

The biological significance of PAK6 is distinct and comparatively less explored in chemokine-focused PD transcriptomic studies. Unlike CCL2, PAK6 is not a canonical inflammatory cytokine or chemokine. Instead, it is a serine/threonine kinase with emerging relevance to neuronal signaling, cytoskeletal regulation, and PD-related LRRK2 biology [40]. Prior mechanistic work has shown that PAK6 phosphorylates 14-3-3γ, thereby regulating LRRK2 phosphorylation and rescuing pathogenic G2019S LRRK2-associated neurite shortening in neuronal systems [17]. Additional studies further demonstrated that PAK6-mediated phosphorylation of PPP2R2C regulates recruitment of the PP2A machinery to the LRRK2 complex, highlighting another layer of LRRK2 phosphoregulation [18]. A recent clinical study also reported altered plasma PAK6 levels in both sporadic and LRRK2-linked PD, suggesting translational relevance beyond mechanistic cell biology [41]. In this context, the emergence of PAK6 from an inflammation- and chemokine-associated candidate space suggests that PD-related neuroimmune dysregulation is closely coupled to neuronal regulatory vulnerability, rather than being confined to canonical inflammatory mediators alone. PAK6 therefore broadens the biological interpretation of the present study from immune signaling alone to the interface between neuroimmune remodeling and neuron-associated regulatory programs.

Another notable aspect of our results is that the enrichment analyses converged on pathways related to mitochondrial organization, respiratory electron transport, oxidative phosphorylation, proteasomal processes, and mitophagy. This enrichment pattern is highly consistent with the established pathobiology of PD, in which mitochondrial dysfunction and impaired mitochondrial quality control are central features of dopaminergic neurodegeneration [42,43]. Rather than contradicting the inflammatory findings, these enrichment results suggest that inflammatory and metabolic disturbances are closely intertwined. In this framework, chemokine-associated signaling may contribute to a hostile extracellular milieu, whereas mitochondrial and proteostatic failure may define the intracellular susceptibility of vulnerable neural populations. The combination of CCL2 and PAK6 is therefore biologically interesting: one candidate is more readily interpreted within an inflammatory chemokine axis, whereas the other points toward neuronal signaling and LRRK2-related regulatory mechanisms. This duality may better reflect the multifactorial nature of PD than a biomarker set confined to cytokine-related genes alone [37,38,43].

Our immune-signature and single-nucleus analyses further strengthen this interpretation. Although transcriptome-based immune deconvolution does not provide the same resolution as direct histology or flow cytometry, it can still reveal disease-associated shifts in inflammatory states and their relationship to candidate genes [3]. This limitation is particularly relevant for the bulk transcriptomic cohorts used here. Accordingly, the ssGSEA results should be interpreted as transcriptome-derived immune-state signatures rather than direct measurements of immune-cell abundance or tissue infiltration [37,38]. In parallel, single-nucleus transcriptomic mapping provided an important layer of biological context by helping distinguish the likely cellular sources of the final candidates. This step is especially valuable in PD, where bulk substantia nigra transcriptomes reflect a composite signal shaped by neuronal loss, reactive glia, vascular components, and immune-related transcriptional changes.

Several limitations should nevertheless be acknowledged. First, the discovery cohort was still based on GSE20141, which was also used in the study by Li et al., and therefore the initial signal space partly overlaps with previous work [20]. Second, the discovery cohort GSE20141 is based on microarray profiling of substantia nigra samples, whereas the validation cohort GSE114517 represents bulk substantia nigra RNA-seq [20,25]. Concordant genes such as CCL2 and PAK6 may therefore reflect disease-associated signals that survive differences in both platform and tissue sampling, whereas non-validated candidates may be more context dependent. Third, the immune landscape was inferred computationally rather than measured experimentally, so these results should be interpreted as transcriptomic immune signatures rather than direct cell counts. Fourth, the snRNA-seq analyses were used primarily for biological contextualization and do not by themselves establish mechanism or causal direction [21,22,25]. Finally, the roles of CCL2 and PAK6 in PD cannot be resolved by bioinformatics alone and require orthogonal validation at the protein, cellular, and functional levels [17,18,41,44].

Overall, the present study prioritizes CCL2 and PAK6 as two candidate molecules that capture complementary dimensions of PD biology: a chemokine-associated inflammatory axis and a neuron-associated regulatory axis. More broadly, our results support a model in which PD-related neuroinflammation is coupled to mitochondrial and cellular vulnerability programs rather than existing as an isolated inflammatory process. Future studies will be needed to determine whether CCL2 and PAK6 primarily reflect disease-associated transcriptional states, whether they have protein-level or biofluid relevance, and whether either candidate also contributes functionally to PD pathogenesis [17,18,20,25,41].

## 5. Conclusions

In conclusion, our integrative analysis prioritized CCL2 and PAK6 as candidate molecules associated with PD-related inflammatory and chemokine-linked transcriptional alterations in the substantia nigra. Cross-platform support from the external validation cohort, together with single-nucleus transcriptomic mapping, further suggested that these two candidates reflect biologically distinct but potentially complementary dimensions of PD pathology: CCL2 was preferentially associated with astrocytes, whereas PAK6 was more strongly associated with neuronal populations, particularly OTX2-positive ventral midbrain neurons. More broadly, the present study supports the view that PD-related neuroinflammation should not be interpreted as a single inflammatory axis alone, but rather as a transcriptional landscape shaped by interactions among chemokine-associated immune remodeling, mitochondrial dysfunction, and cell-type-specific vulnerability.

At the same time, the present study should be interpreted within the boundaries of a computational, public-dataset-based analysis. The discovery-stage screening was exploratory, the machine learning results require continued evaluation of robustness across datasets and analytical settings, and the in silico perturbation analysis provides predictive, hypothesis-generating rather than causal evidence. Accordingly, future studies will be needed to validate CCL2 and PAK6 in independent clinical and experimental cohorts, to determine their protein-level and biofluid relevance, and to clarify whether they serve primarily as biomarkers of disease-associated transcriptional states or also as functional contributors to PD pathogenesis.

## Data Availability

The datasets analyzed in this study are publicly available in the Gene Expression Omnibus (GEO) under accession numbers GSE20141, GSE114517, GSE148434, GSE253462, and GSE253975.

## Supplementary Materials

The following supporting information can be downloaded at: https://www.mdpi.com/article/10.3390/brainsci16050463/s1. Figure S1: Per-seed hit map of CCL2 across 0–100 random seeds in Elastic Net, SVM-RFE, and random forest. Figure S2: Additional external support for CCL2 from GSE148434 and GSE253975. Figure S3: Detailed single-nucleus characterization of the final prioritized candidates. Figure S4: CellChat analysis of differential ligand–receptor interactions between control and PD conditions.

## References

[1] Morris H.R., Spillantini M.G., Sue C.M., Williams-Gray C.H. (2024). The pathogenesis of Parkinson’s disease. Lancet.

[2] Bloem B.R., Okun M.S., Klein C. (2021). Parkinson’s disease. Lancet.

[3] Poewe W., Seppi K., Tanner C.M., Halliday G.M., Brundin P., Volkmann J., Schrag A.E., Lang A.E. (2017). Parkinson disease. Nat. Rev. Dis. Primers.

[4] Jurcau A., Andronie-Cioara F.L., Nistor-Cseppento D.C., Pascalau N., Rus M., Vasca E., Jurcau M.C. (2023). The Involvement of Neuroinflammation in the Onset and Progression of Parkinson’s Disease. Int. J. Mol. Sci..

[5] Pajares M., Rojo A.I., Manda G., Boscá L., Cuadrado A. (2020). Inflammation in Parkinson’s Disease: Mechanisms and Therapeutic Implications. Cells.

[6] Roodveldt C., Bernardino L., Oztop-Cakmak O., Dragic M., Fladmark K.E., Ertan S., Aktas B., Pita C., Ciglar L., Garraux G. (2024). The immune system in Parkinson’s disease: What we know so far. Brain.

[7] Chen K., Wang H., Ilyas I., Mahmood A., Hou L. (2023). Microglia and Astrocytes Dysfunction and Key Neuroinflammation-Based Biomarkers in Parkinson’s Disease. Brain Sci..

[8] Isik S., Yeman Kiyak B., Akbayir R., Seyhali R., Arpaci T. (2023). Microglia Mediated Neuroinflammation in Parkinson’s Disease. Cells.

[9] Voet S., Prinz M., van Loo G. (2019). Microglia in Central Nervous System Inflammation and Multiple Sclerosis Pathology. Trends Mol. Med..

[10] Brandebura A.N., Paumier A., Onur T.S., Allen N.J. (2023). Astrocyte contribution to dysfunction, risk and progression in neurodegenerative disorders. Nat. Rev. Neurosci..

[11] Patani R., Hardingham G.E., Liddelow S.A. (2023). Functional roles of reactive astrocytes in neuroinflammation and neurodegeneration. Nat. Rev. Neurol..

[12] Kim C., Ho D.H., Suk J.E., You S., Michael S., Kang J., Joong Lee S., Masliah E., Hwang D., Lee H.J. (2013). Neuron-released oligomeric α-synuclein is an endogenous agonist of TLR2 for paracrine activation of microglia. Nat. Commun..

[13] Béraud D., Maguire-Zeiss K.A. (2012). Misfolded α-synuclein and Toll-like receptors: Therapeutic targets for Parkinson’s disease. Park. Relat. Disord..

[14] Fellner L., Irschick R., Schanda K., Reindl M., Klimaschewski L., Poewe W., Wenning G.K., Stefanova N. (2013). Toll-like receptor 4 is required for α-synuclein dependent activation of microglia and astroglia. Glia.

[15] Shen R., Lin S., He L., Zhu X., Zhou Z., Chen S., Wang Y., Ding J. (2019). Association of Two Polymorphisms in CCL2 With Parkinson’s Disease: A Case-Control Study. Front. Neurol..

[16] Harms A.S., Thome A.D., Yan Z., Schonhoff A.M., Williams G.P., Li X., Liu Y., Qin H., Benveniste E.N., Standaert D.G. (2018). Peripheral monocyte entry is required for alpha-Synuclein induced inflammation and Neurodegeneration in a model of Parkinson disease. Exp. Neurol..

[17] Civiero L., Cogo S., Kiekens A., Morganti C., Tessari I., Lobbestael E., Baekelandt V., Taymans J.M., Chartier-Harlin M.C., Franchin C. (2017). PAK6 Phosphorylates 14-3-3γ to Regulate Steady State Phosphorylation of LRRK2. Front. Mol. Neurosci..

[18] Iannotta L., Emanuele M., Favetta G., Tombesi G., Vandewynckel L., Lara Ordóñez A.J., Saliou J.M., Drouyer M., Sibran W., Civiero L. (2023). PAK6-mediated phosphorylation of PPP2R2C regulates LRRK2-PP2A complex formation. Front. Mol. Neurosci..

[19] Krokidis M.G. (2019). Identification of biomarkers associated with Parkinson’s disease by gene expression profiling studies and bioinformatics analysis. AIMS Neurosci..

[20] Li Y., Jia W., Chen C., Chen C., Chen J., Yang X., Liu P. (2025). Identification of biomarkers associated with inflammatory response in Parkinson’s disease by bioinformatics and machine learning. PLoS ONE.

[21] Ma S.X., Lim S.B. (2021). Single-Cell RNA Sequencing in Parkinson’s Disease. Biomedicines.

[22] Smajić S., Prada-Medina C.A., Landoulsi Z., Ghelfi J., Delcambre S., Dietrich C., Jarazo J., Henck J., Balachandran S., Pachchek S. (2022). Single-cell sequencing of human midbrain reveals glial activation and a Parkinson-specific neuronal state. Brain.

[23] Edgar R., Domrachev M., Lash A.E. (2002). Gene Expression Omnibus: NCBI gene expression and hybridization array data repository. Nucleic Acids Res..

[24] Ritchie M.E., Phipson B., Wu D., Hu Y., Law C.W., Shi W., Smyth G.K. (2015). limma powers differential expression analyses for RNA-sequencing and microarray studies. Nucleic Acids Res..

[25] Simchovitz A., Hanan M., Yayon N., Lee S., Bennett E.R., Greenberg D.S., Kadener S., Soreq H. (2020). A lncRNA survey finds increases in neuroprotective LINC-PINT in Parkinson’s disease substantia nigra. Aging Cell.

[26] Liberzon A., Birger C., Thorvaldsdóttir H., Ghandi M., Mesirov J.P., Tamayo P. (2015). The Molecular Signatures Database (MSigDB) hallmark gene set collection. Cell Syst..

[27] Szklarczyk D., Gable A.L., Nastou K.C., Lyon D., Kirsch R., Pyysalo S., Doncheva N.T., Legeay M., Fang T., Bork P. (2021). The STRING database in 2021: Customizable protein-protein networks, and functional characterization of user-uploaded gene/measurement sets. Nucleic Acids Res..

[28] Shannon P., Markiel A., Ozier O., Baliga N.S., Wang J.T., Ramage D., Amin N., Schwikowski B., Ideker T. (2003). Cytoscape: A software environment for integrated models of biomolecular interaction networks. Genome Res..

[29] Yu G., Wang L.G., Han Y., He Q.Y. (2012). clusterProfiler: An R package for comparing biological themes among gene clusters. Omics.

[30] Hänzelmann S., Castelo R., Guinney J. (2013). GSVA: Gene set variation analysis for microarray and RNA-seq data. BMC Bioinform..

[31] Huang S., Cai N., Pacheco P.P., Narrandes S., Wang Y., Xu W. (2018). Applications of Support Vector Machine (SVM) Learning in Cancer Genomics. Cancer Genom. Proteom..

[32] Hu J., Szymczak S. (2023). A review on longitudinal data analysis with random forest. Brief. Bioinform..

[33] Subramanian A., Tamayo P., Mootha V.K., Mukherjee S., Ebert B.L., Gillette M.A., Paulovich A., Pomeroy S.L., Golub T.R., Lander E.S. (2005). Gene set enrichment analysis: A knowledge-based approach for interpreting genome-wide expression profiles. Proc. Natl. Acad. Sci. USA.

[34] Warde-Farley D., Donaldson S.L., Comes O., Zuberi K., Badrawi R., Chao P., Franz M., Grouios C., Kazi F., Lopes C.T. (2010). The GeneMANIA prediction server: Biological network integration for gene prioritization and predicting gene function. Nucleic Acids Res..

[35] Stuart T., Butler A., Hoffman P., Hafemeister C., Papalexi E., Mauck W.M., Hao Y., Stoeckius M., Smibert P., Satija R. (2019). Comprehensive Integration of Single-Cell Data. Cell.

[36] Armstrong G., Martino C., Rahman G., Gonzalez A., Vázquez-Baeza Y., Mishne G., Knight R. (2021). Uniform Manifold Approximation and Projection (UMAP) Reveals Composite Patterns and Resolves Visualization Artifacts in Microbiome Data. mSystems.

[37] Tansey M.G., Wallings R.L., Houser M.C., Herrick M.K., Keating C.E., Joers V. (2022). Inflammation and immune dysfunction in Parkinson disease. Nat. Rev. Immunol..

[38] Araújo B., Caridade-Silva R., Soares-Guedes C., Martins-Macedo J., Gomes E.D., Monteiro S., Teixeira F.G. (2022). Neuroinflammation and Parkinson’s Disease-From Neurodegeneration to Therapeutic Opportunities. Cells.

[39] Xiromerisiou G., Marogianni C., Lampropoulos I.C., Dardiotis E., Speletas M., Ntavaroukas P., Androutsopoulou A., Kalala F., Grigoriadis N., Papoutsopoulou S. (2022). Peripheral Inflammatory Markers TNF-α and CCL2 Revisited: Association with Parkinson’s Disease Severity. Int. J. Mol. Sci..

[40] Zhang K., Wang Y., Fan T., Zeng C., Sun Z.S. (2022). The p21-activated kinases in neural cytoskeletal remodeling and related neurological disorders. Protein Cell.

[41] Giusto E., Maistrello L., Iannotta L., Giusti V., Iovino L., Bandopadhyay R., Antonini A., Bubacco L., Barresi R., Plotegher N. (2024). Prospective Role of PAK6 and 14-3-3γ as Biomarkers for Parkinson’s Disease. J. Park. Dis..

[42] Xiao B., Kuruvilla J., Tan E.K. (2022). Mitophagy and reactive oxygen species interplay in Parkinson’s disease. npj Park. Dis..

[43] Klemmensen M.M., Borrowman S.H., Pearce C., Pyles B., Chandra B. (2024). Mitochondrial dysfunction in neurodegenerative disorders. Neurotherapeutics.

[44] Iannotta L., Fasiczka R., Favetta G., Zhao Y., Giusto E., Dall’Ara E., Wei J., Ho F.Y., Ciriani C., Cogo S. (2024). PAK6 rescues pathogenic LRRK2-mediated ciliogenesis and centrosomal cohesion defects in a mutation-specific manner. Cell Death Dis..

