# Supplementary material for: CCL2 and PAK6 as Candidate Biomarkers of Neuroinflammation in Parkinson’s Disease: An Integrated Machine Learning and Single-Nucleus Transcriptomic Study
Source: Brain Sci. 2026 Apr 25;16(5):463. doi: 10.3390/brainsci16050463 (PMC13204684; doi:10.3390/brainsci16050463)
Supplement: Supplementary file 1 [file brainsci-16-00463-s001.zip › brainsci-4267984-supplementary.pdf]

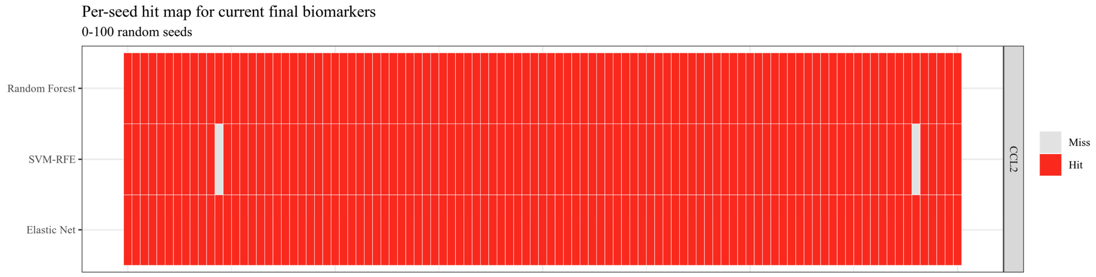

Supplementary Figure S1. Per-seed hit map of CCL2 across 0–100 random seeds in Elastic Net, support vector machine-recursive feature elimination (SVM-RFE), and random forest. Red indicates that CCL2 was retained by the corresponding method under a given seed, whereas gray indicates non-selection.

(A)

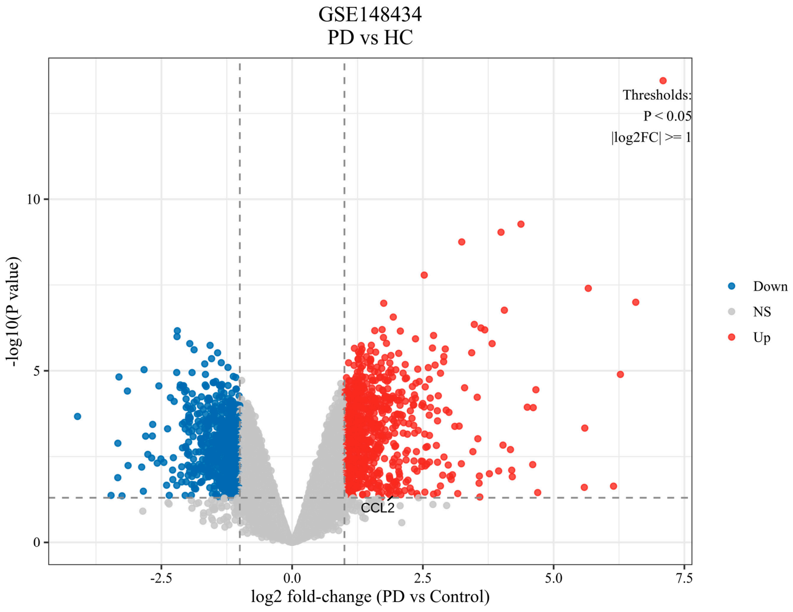

(B)

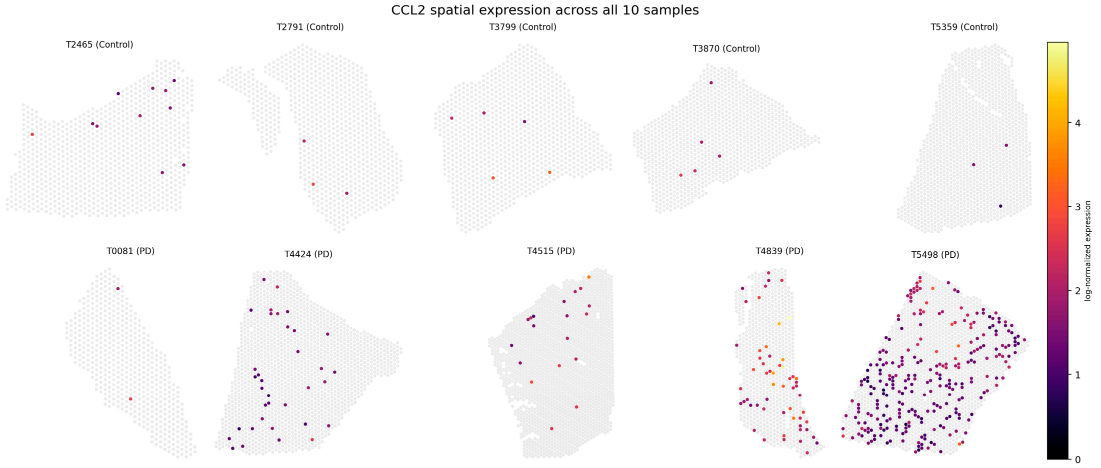

Supplementary Figure S2. Additional external support for CCL2 from GSE148434 and GSE253975. (A) Volcano plot showing differential-expression patterns between Parkinson's disease (PD) and healthy control (HC) samples in the independent GSE148434 dataset. Red dots indicate upregulated genes, blue dots indicate downregulated genes, and gray dots indicate non-significant genes. Vertical dashed lines indicate ∣log2FC∣ = 1, and the horizontal dashed line indicates $P = 0.05$. CCL2 is highlighted to illustrate its PD-upregulated position in this additional external dataset. (B) Spatial transcriptomic visualization of CCL2 expression across all 10 substantia nigra samples from GSE253975, including control and PD cases. Colored spots indicate normalized CCL2 expression, with warmer colors representing higher expression levels. This panel provides spatial support for disease-associated CCL2 expression in human PD substantia nigra.

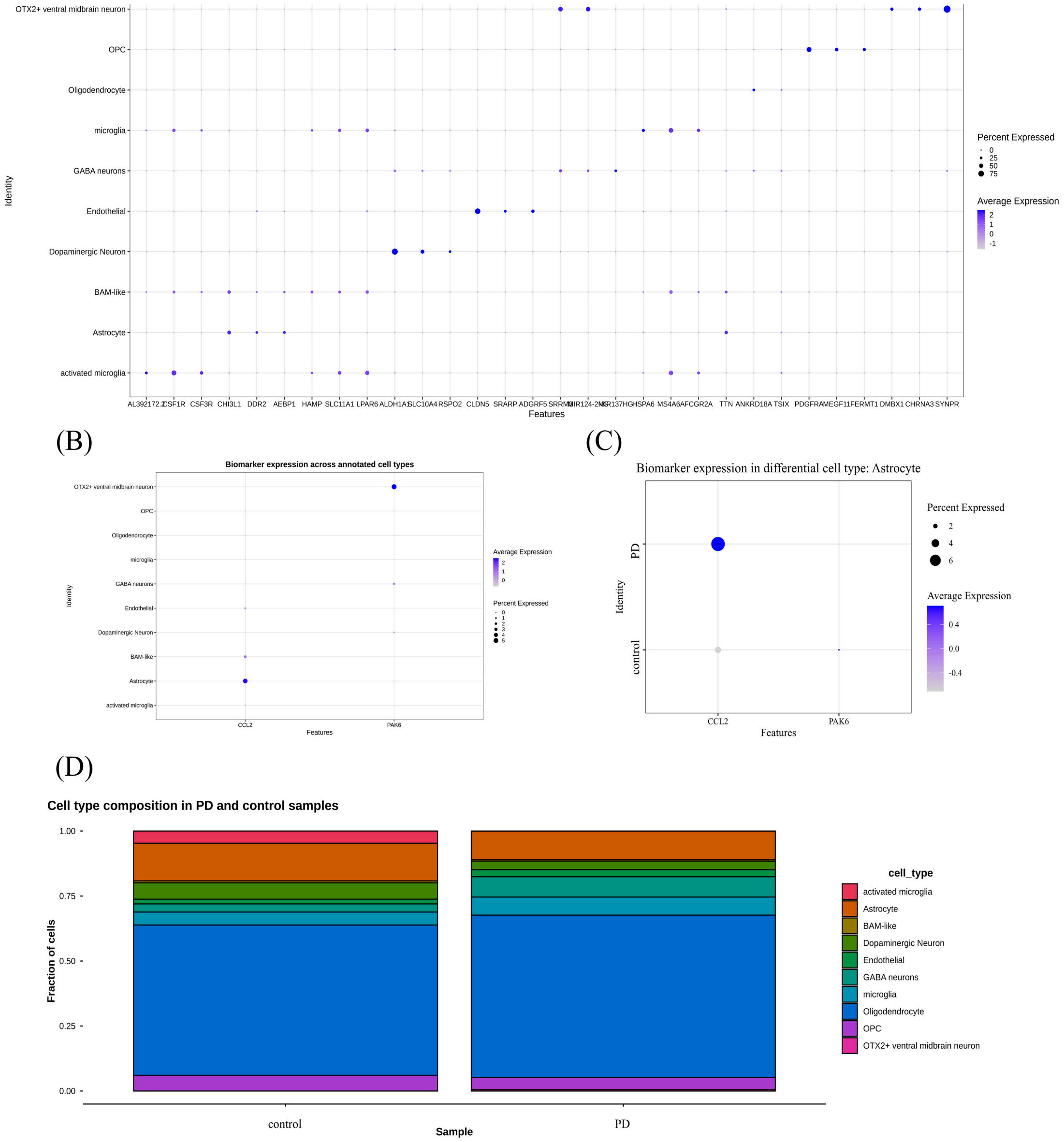

Supplementary Figure S3. Detailed single-nucleus characterization of the final prioritized candidates. (A) Dot plot of representative marker genes across annotated cell types. (B) Dot plot showing the distribution of CCL2 and PAK6 across annotated cell populations. (C) Dot plot comparing CCL2 and PAK6 expression in astrocytes between control and Parkinson's disease (PD) groups. (D) Stacked bar plots showing cell-type composition in control and PD samples.

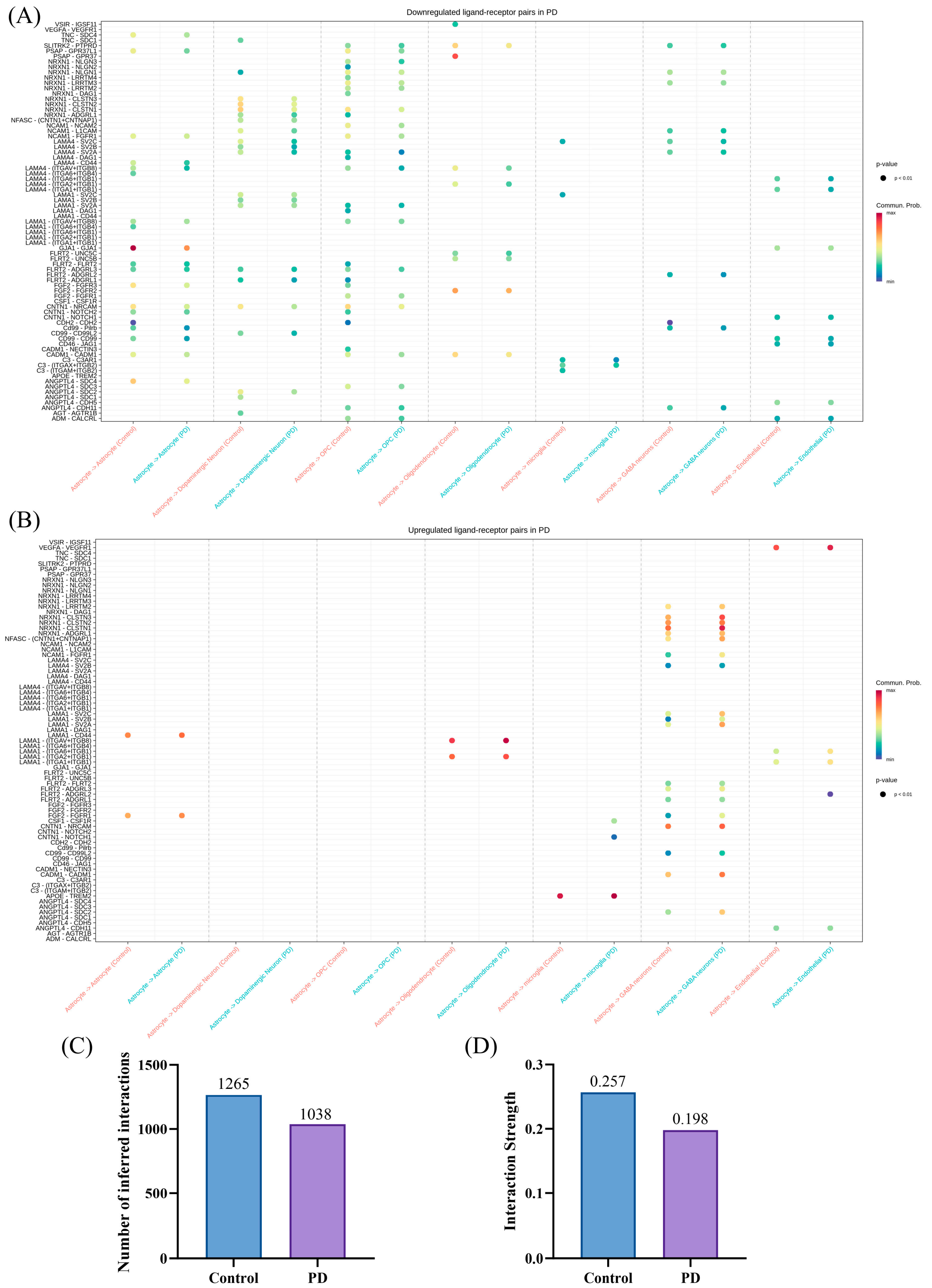

Supplementary Figure S4. CellChat analysis of differential ligand–receptor interactions between control and PD conditions. (A) Bubble plot showing ligand–receptor pairs relatively downregulated in PD, with emphasis on astrocyte-associated interactions. (B) Bubble plot showing ligand–receptor pairs relatively upregulated in PD. (C) Summary comparison of overall interaction counts between control and PD conditions. (D) Summary comparison of overall interaction weights between control and PD conditions.
